# Health and Athletics

**Published:** 1895-08-17

**Authors:** 


### Health and Athletics.

Sport rules the world. Whether in summer or in winter games appropriate to the season are always in full swing. Every newspaper is crowded with their reports; papers, indeed, are printed which would seem to scorn allusion to anything else, and whether it be in the drawing-room, the office, or the stable, or even as a way to the heart of a porter or a guard, the subject of athletics is an open sesame, a card which it is always safe to play. What, then, is the object of all this exercise and training, this excitement on the one hand, this self-denial on the other? Is it that a healthy mind may in a healthy body be maintained? We fear not, but that it rather is the outcome of a spirit of emulation, a striving for the mastery, an endeavour at all hazards to be first. The excuse everywhere made for the extravagant amount of time spent by young people over athletic games is—that it all conduces to health. Schoolmasters point to their healthy boys and lay their good looks to the credit of compulsory games, even morality is associated with athletics, it being held that there is something shaky about a lad who shirks his football, and college tutors, much as they bemoan the loss of time, admit the finer physique, the wider chest, the greater vigour of those whose college course is one long training in athletics. Yet in all this there is a fallacy. The schools which stand well in their matches attract the boys of stronger build, and, what is perhaps more important, attract the sons of men who have played themselves in youth. Thus the class of boys drawn to a school where athletics are a feature are often boys of better build and breed than those who go to places of less muscular reputation. A process of natural selection thus takes place by means of original taste, which is even more effective than many a medical entrance examination might prove.

Still more does this process of natural selection sort out the men who go to different colleges. Thus, although it is undeniable that the school which is strong in games shows the ruddiest boys, and boys the best set up, that the athletic college shows the finest men, and that in a given college, there is a marked difference between the sporting and the reading set, if taken as a whole, although marked exceptions are often seen; yet the credit of this must not all be put down to the athletics, but rather be attributed in large degree to the original vigour with which the individuals were blessed.

For all this the popular verdict is that athletics lead to health, and so strongly is this opinion held, and so ready are many people to condone much backwardness in other things if young men do but come out well in sports, on the ground that they are laying the foundations of that *corpus sanum* in which, it is hoped, the *mens sana* will ultimately thrive, that it is worth while to draw attention to the fact that athletics must be used in quite a different manner, when employed as a means to health, to that in which they are commonly indulged in, and that striving to excel, struggling to be first, putting out the utmost energy, and straining every nerve, for honour, applause, and "cups," would have no part in such hygienic exercises. The broad line of distinction between the two kinds or degrees of athletic exercise, the one of which leads to health and the other, not perhaps to immediate disease, for there is wonderful elasticity and adaptability in the human frame, but ultimately to a premature muscular or nervous failure, lies in the fact that exercise to be good and healthful should not disturb the proportionate vigour of the different organs, and that any form of training or athletics by which this proportion is deranged will sooner or later lead to trouble.

Sir B. W. Richardson has recently pointed out that health involves a due balance between all the functions, and that the excessive or prolonged strain necessitated by some of the athletic feats and record-breaking efforts which are now so commonly undertaken, often with but small idea of their ultimate harmfulness, has the effect of disturbing this proper balance and thus causing disease.

A rough and popular view of the effect of our exercise would be that, however "tired" one might become, or any over-exerted muscle might become, rest would pull all right; and for such muscles as can rest the popular view is not far wrong. But there are certain vital organs which do not rest, and do not easily, after great exertion, subside to quietness. The heart, for example, under the influence of violent exercise and hard training, develops and hypertrophies, grows larger and stronger, and at each beat puts out more energy, and thus is able to supply the labouring muscles with more blood. Up to a certain point this is good—it is but a part of the harmonious development of the whole body—beyond this it is bad, for while the external muscles, of which alone the athlete thinks, lie fallow and recuperate, the heart which cannot rest does not quickly revert to its former state, but with clumsy vigour goes on driving the blood into the tired and overstretched blood-vessels, denying to them also the rest which it cannot obtain itself. It is here, in the heart and blood-vessel, that the violent and especially the prolonged exercise now so commonly indulged in ultimately tells, and grand as it may be to have a heart so powerful that it will go on for twenty-four hours at a stretch pumping blood so freely to every part of the body, that every muscle can keep in full activity all the time, as in some long cycle rides, still such a heart is not wanted for the ordinary requirements of the body, disproportion has been reached, and that due proportion which means health is not easily regained.

All this we fear is a dull doctrine, for we know that, common as is the talk about the healthy tendencies of athletic sports, the question of health hardly enters into the minds of those who most vigorously indulge in them. It is something, however, to tear down the veil and insist on the true relationship of health and athletics. Writing on this subject, Sir B. W. Richardson says: "A man is said to 'throw himself heart and soul into his work.' Such a man is in danger." And he also says, "The grand lesson we have to learn is to cultivate health; to overcome the excessive desire of competition; to be strong and skilful and enduring, but not ambitious after the manner of children who wish to be at the head or nowhere."

We fear, however, that if those who indulge in athletic sports, so greatly to the admiration of a gate-money paying public, were to take this lesson to heart, and so play as only to "cultivate health," much of the popular interest in such games would fall away. Athletics may be so used as to be health giving; whether they are so used at the present is quite another question.

